# Sewer Gas

**Published:** 1875-12

**Authors:** 


### SEWER GAS.

The question has been asked with renewed emphasis of late, can nothing be done that will effectually prevent defective house-drainage, imperfect soil and waste pipes, and the danger to health arising from the escape of noxious gases in dwelling-houses. An experienced plumber declares that the fault lies largely with his craft. In a city like New York, where water conveniences are so multiplied, and which, if properly constructed, conduce so much to the comfort and health of the people, there should be the greatest care exercised in having these conveniences constructed upon the soundest scientific theory, combined with the most practical utility. A dwelling-house fitted up with water-closets, baths, wash-basins, &c., may be a luxury of enjoyment to the occupants, and also may be a perfect pest-house, if the work be improperly or ignorantly done.

To answer the questions propounded in the beginning of this article, we would say that there is no difficulty in arranging the sewer, soil and waste pipes in a house so that at no time will they emit foul air or sewer gas. To do this the following plan must be adopted, and rigidly carried out. The main sewer-pipe in the cellar, which receives all the waste water of the house, should be laid with a good and regular descent from the rear of the house to the sewer in the street. The joints in this pipe must be made absolutely air and water tight. In this pipe there should be no trap. This pipe should receive the rain water leader, and the nearer the leader approaches in size to that of the sewer-pipe the better the ventilation of the latter; and the rapid escape of sewer gas through the leader to the atmosphere above the roof will be materially assisted.

The soil-pipe from water-closets, and wash-pipes from wash basins in the upper part of the house should run separately and perpendicularly to the sewer-pipe in the cellar, and be connected therewith at that point by a trap on each line at least one size larger than the soil or waste pipe itself. These pipes should in all cases be extended, full size, to a height say two feet above the roof and finished with a properly constructed ventilating cap.

All fixtures should be separately trapped as close to the apparatus as the nature of its construction will admit. When lateral branches of waste pipe of any considerable length are required, they should be a little larger than the traps attached to them. The reason for this is obvious. The water that passes through the trap cannot fill the pipe by reason of its greater capacity, and a solid column of water cannot be formed so as to create a syphon, and thus empty the trap.

In many of the houses in this city, which have been built on speculation, the plumbing has generally been auctioned off to the lowest bidder, regardless of his capacity or responsibility; consequently the work has been done according to the price paid—or it has been done by parties profoundly innocent of any but the most incipient knowledge of their business. It is in these houses that so much trouble arises from the escape of sewer gas. In very many of the houses which have been built fifteen or twenty years, the soil and waste pipes are very old and need renewing. The old style of water-closet, too, is a productive source of annoyance from the fact that the receiver is a receptable of foul air which, to a greater or lesser extent, escapes into the apartment when the pan is dropped. The basins attached to these closets will in time become impregnated with a powerful acid which penetrates through the glaze upon the surface of the basin, and which no washing will effectually cleanse. Experience in correcting the evils herein enumerated has done considerable, and where the plan above described has been thoroughly carried out, the remedy has been successful; and we have no hesitation in saying that a house with its soil and waste pipes so constructed, with the use of the improved water-closet, will be perfectly free from sewer-gas and any of those disagreeable odors which make so many of even our first-class houses abodes of lurking disease and hidden pestilence. A system of ventilating the seats of water-closets by connecting a pipe with a hot-air flue, such as the flue of the kitchen chimney, or a flue heated by means of gas-burners, and extending the pipe under the seat of the water-closets, has been frequently tried here and found very effectual. And if, where houses are being erected, iron pipe flues could be constructed within the flue of the kitchen chimney, with branches extending to the water-closets on each floor, it would be found to be one of the most efficient means of drawing off from the water-closet seat, the effluvia almost inseparably connected with its use.

---

Dr. Trelat, Dr. Moreau, and other well-known French physicians have recently made a report on lunacy in France. Madness falls heaviest in Paris on the artisans and cooks. Next to them is the trading class. Insanity is not frequent in men belonging to liberal professions, and the proportion is lowest among gardeners and spade laborers. The doctors signing the report do not take into account the injurious agency of leaded tin, which was to a certain extent countenanced by the Government in 1862, when the Ministry of War allowed hardware contractors to use six per cent. of lead in the tinning of military camp kettles and other culinary vessels.

